# Spontaneous Resolution of the Fe(C_2_O_4_)_3_ Anion and Inclusion of Chiral Guest Molecules in BEDT-TTF Radical-Cation Salts

**DOI:** 10.1021/acs.inorgchem.4c04622

**Published:** 2025-01-07

**Authors:** Joseph O. Ogar, John D. Wallis, Toby J. Blundell, Elizabeth K. Rusbridge, Alex Mantle, Hiroki Akutsu, Yasuhiro Nakazawa, Shusaku Imajo, Lee Martin

**Affiliations:** † School of Science and Technology, Nottingham Trent University, Nottingham, Clifton Lane NG11 8NS, U.K.; ‡ Department of Chemistry, Graduate School of Science, Osaka University, 1-1 Machikaneyama, Toyonaka, Osaka 560-0043, Japan; § Institute for Solid State Physics, University of Tokyo, Kashiwa 277-8581, Japan

## Abstract

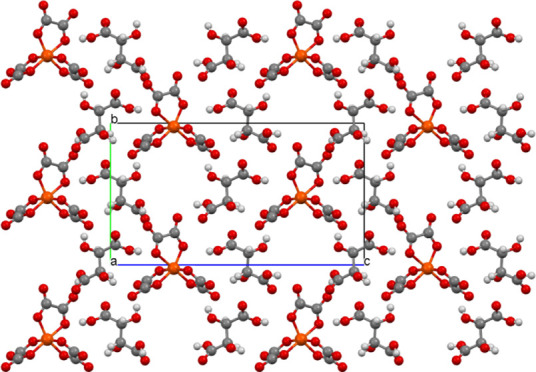

We report the synthesis of three radical-cation salts of BEDT-TTF from racemic tris(oxalato)ferrate by electrocrystallization in the presence of chiral molecules. In the presence of enantiopure l-(+)-tartaric acid, we observe spontaneous resolution of the labile tris(oxalato)ferrate anion to produce the chiral radical-cation salt α-(BEDT-TTF)_5_[Δ-Fe(C_2_O_4_)_3_].[l-(+)-tartaric acid]_2_ which contains only the Δ enantiomer of Fe(C_2_O_4_)_3_. Using enantiopure (*R*)-(−)-3-hydroxytetrahydrofuran as an additive gives α-(BEDT-TTF)_12_[Fe(oxalate)_3_]_2_.(H_2_O)_16_.ethanol.(*R*)-(−)-3-hydroxytetrahydrofuran, which includes the chiral guest and contains a racemic mixture of Λ and Δ enantiomers of Fe(C_2_O_4_)_3_. Enantiopure (*S*)-phenyloxirane is hydrolyzed to racemic 1-phenylethane-1,2-diol to produce racemic β”-β”-(BEDT-TTF)_4_(H_2_O/H_3_O)Fe(C_2_O_4_)_3_.1-phenylethane-1,2-diol.

## Abstract 2

The inclusion of enantiopure guests has produced three new radical-cation salts from BEDT-TTF with racemic tris(oxalato)ferrate. With enantiopure l-(+)-tartaric acid resolution of the labile tris(oxalato)ferrate anion occurs to produce the chiral radical-cation salt α-(BEDT-TTF)_5_[Δ-Fe(C_2_O_4_)_3_].[l-(+)-tartaric acid]_2_ which contains only the Δ enantiomer of Fe(C_2_O_4_)_3_. Using enantiopure (*R*)-(−)-3-hydroxytetrahydrofuran gives α-(BEDT-TTF)_12_[Fe(oxalate)_3_]_2_.(H_2_O)_16_.ethanol.(*R*)-(−)-3-hydroxytetrahydrofuran, which includes the chiral guest and contains a racemic mixture of Λ- and Δ-Fe(C_2_O_4_)_3_. Enantiopure (*S*)-phenyloxirane is hydrolyzed to racemic 1-phenylethane-1,2-diol to produce racemic β”-β”-(BEDT-TTF)_4_(H_2_O/H_3_O)Fe(C_2_O_4_)_3_.1-phenylethane-1,2-diol.

## Introduction

TTF and BEDT-TTF radical-cation salts offer the possibility of combining multiple properties together in the same material that are not found together in nature.^1^ They consist of alternating conducting TTF or BEDT-TTF donor layers and insulating anion layers. Of particular interest in recent years has been their ability to combine chirality together with electrical conductivity.^2^ These salts offer great potential for comparing the conducting behavior of both enantiomeric forms and also the racemate to investigate electrical magnetochiral anisotropy (eMChA), where the electrical resistivity in an applied magnetic field differs depending upon the handedness of the material. This has been observed in carbon nanotubes^3^ and bismuth helices.^4^ Recently, superconductors with structural chirality have also been reported.^5^

eMChA has also been reported in radical-cation salts of the donors (*S*,*S*)- and (*R*,*R*)-(DM-EDT-TTF)_2_ClO_4_ which are metallic down to 40 K and crystallize in enantiomorphic space groups *P*6_2_22 and *P*6_4_22 (DM-EDT-TTF = dimethyl-ethylenedithio-tetrathiafulvalene).^6^ A number of enantiopure donors have been synthesized since the first TTF chiral donor, tetramethyl-(*S*,*S*,*S*,*S*)-BEDT-TTF.^7^ Recently, a 4:1 salt of the chiral donor (1’*R*,5*S*)-*N*-(1′-phenylethyl)(BEDT-TTF)acetamide with TCNQ was reported to be a chiral metal down to 4.2 K with a room-temperature transition to an insulating state above 283 K (TCNQ = tetracyanoquinodimethane).^8^

Using an enantiopure donor molecule^2^ is one of three methods that have been used to synthesize chiral radical-cation salts, the others being the use of an enantiopure anion (Sb_2_(l-tartrate)_2_^9^ or TRISPHAT (tris(tetrachlorocatecholato)phosphate)^10^ or an enantiopure solvent.^11^ Racemic spiroborate anions with malate^12^ or mandelate^13^ ligands have been shown to spontaneously resolve with only a single enantiomer, or a single pair of diastereomers, being included in the radical-cation salt. A salt of BDH-TTP (BDH-TTP = 2,5-bis(1,3-dithiolan-2-ylidene)-1,3,4,6-tetrathiapentalene) incorporating the enantiopure B(2-chloromandelate)_2_^–^ anion from racemic starting spiroborate is metallic down to at least 4.2 K.^14^

**Scheme 1:**
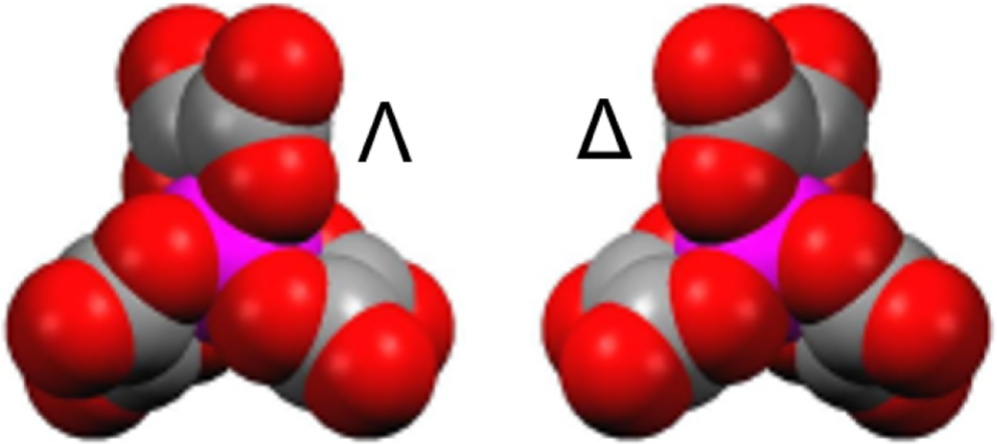
Λ and Δ Forms of Tris(oxalato)ferrate

The most widely used type of racemic anion in radical-cation salts is tris(oxalato)metalate, which has a metal center that can introduce magnetic moments into the anion layer.^15^ These salts contain a racemic mixture of the Δ and Λ enantiomers (Scheme 1) of tris(oxalato)metalate, and the arrangement of the two enantiomers in the lattice can have a profound effect on the donor arrangement and therefore on the electronic resistivity of the salt.^16^ The tris(oxalato)ferrate anion is labile; however, the tris(oxalato)chromate anion has been successfully synthesized in the enantiopure form, but it racemizes in solution before crystals of the BEDT-TTF salt can grow, which produces a salt containing a racemic mixture of the anion.^16^ The use of (*R*)-carvone as the electrocrystallization medium leads to spontaneous resolution of the tris(oxalato)metalate to produce salts containing only a single enantiomer of the anion, but (*R*)-carvone is not included as a guest in the salts.^17^*sec*-Phenethyl alcohol has been used as the electrocrystallization medium and is incorporated into isostructural crystal structures as a guest molecule, leading to isostructural chiral (*S*)- or racemic (*R/S*)- salts, which differ in their electrical resistivities due to the disorder in the racemate compared to the chiral salt.^18^

In this paper, we report for the first time the use of solid chiral additives within the electrocrystallization medium, which leads to three different outcomes: (*S*)-phenyloxirane is hydrolyzed to racemic 1-phenylethane-1,2-diol to give a racemic BEDT-TTF radical-cation salt (**I**). When l-(+)-tartaric acid is included as the guest in the BEDT-TTF radical-cation salt (**II**), there is spontaneous resolution of the tris(oxalato)ferrate anion. The inclusion of (*R*)-(−)-3-hydroxytetrahydrofuran as a guest molecule in the BEDT-TTF radical-cation salt gives a chiral salt (**III**) that contains both Δ and Λ enantiomers of the tris(oxalato)ferrate anion. These chiral guest molecules were selected owing to their size being comparable to the guest molecules which have previously been incorporated into the family of superconducting salts β”-(BEDT-TTF)_4_(H_2_O/H_3_O)Fe(C_2_O_4_)_3_.GUEST. In these salts, the guest molecules are located in the hexagonal cavities within the tris(oxalato)ferrate anion layer.^15−18^

## Results and Discussion

### β”-β”-(BEDT-TTF)_4_(H_2_O/H_3_O)Fe(C_2_O_4_)_3_.1-phenylethane-1,2-diol (I)

**Scheme 2:**
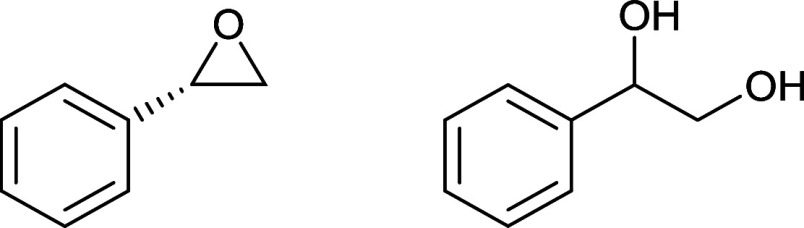
(*S*)-Phenyloxirane (Left) and Racemic 1-Phenylethane-1,2-diol (Right)

The use of chiral (*S*)-phenyloxirane as the solid additive in the electrocrystallization medium produces a bilayered salt, β”-β”-(BEDT-TTF)_4_(H_2_O/H_3_O)Fe(C_2_O_4_)_3_.1-phenylethane-1,2-diol (**I**). Under the conditions of electrocrystallization, the (*S*)-phenyloxirane guest molecule (Scheme 2, left) has hydrolyzed to 1-phenylethane-1,2-diol (Scheme 2, right).^19^ Hydrolysis, over the 8 weeks of the electrocrystallization, catalyzed by acid or Fe^3+^ would take place at the secondary center. If this went via a carbocation, then either enantiomer of the diol could be produced. Water is available due to hydrated ammonium tris(oxalate)ferrate and hygroscopic 18-crown-6 used.

Salt (**I**) crystallizes in the space group *P*1̅ and has an asymmetric unit of four crystallographically independent BEDT-TTF donor molecules, a tris(oxalato)ferrate anion, a 1-phenylethane-1,2-diol molecule, and a H_2_O or H_3_O^+^ molecule.

**Figure 1:**
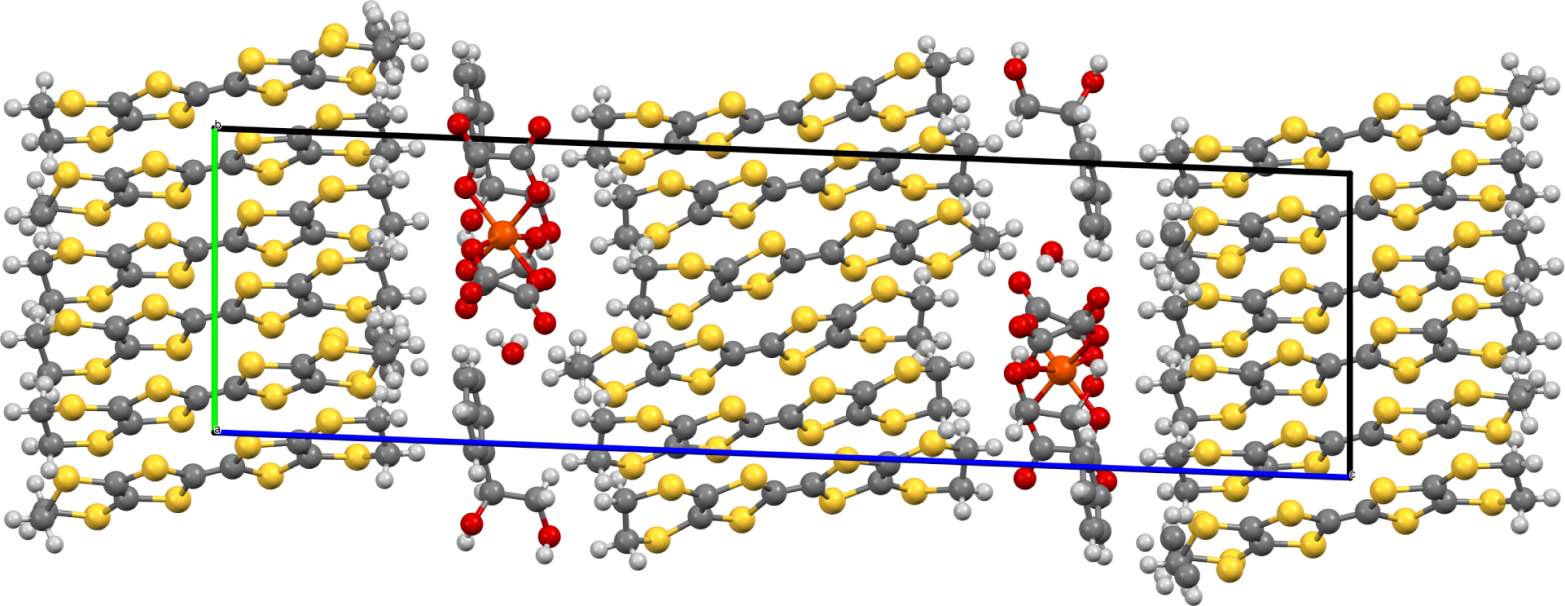
Salt (**I**) viewed down the *a* axis with the *c* axis horizontal showing the two crystallographically unique donor layers and the two anion layers, which are related by a center of symmetry.

**Figure 2:**
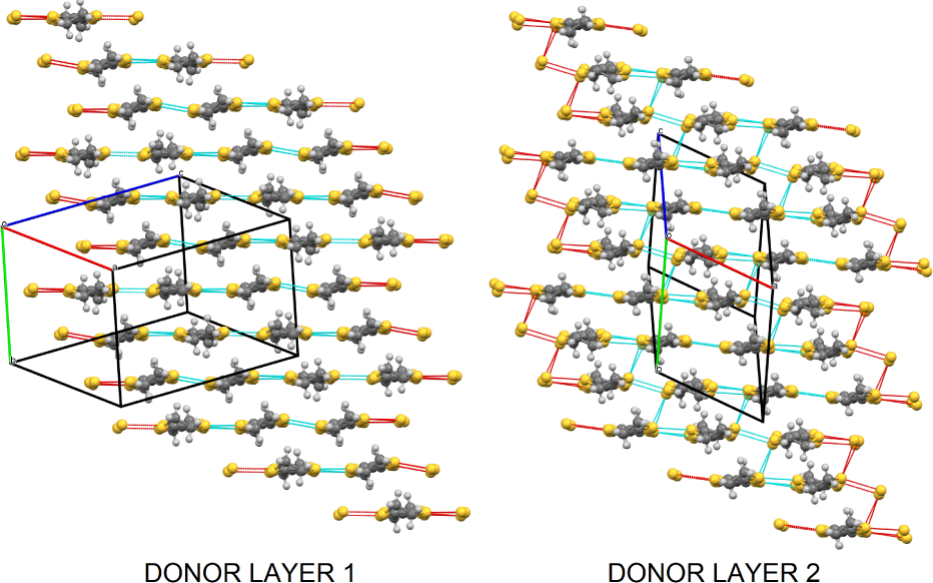
Salt (**I**) donor layers showing the short S···S contacts. Donor layer 1 (left) is on the edge of the unit cell (*c* axis) docking into the anion layers facing the −CH_2_OH groups of the guest molecules. Donor layer 2 (right) is in the center of the unit cell (*c* axis) docking into anion layers and facing the phenyl groups of the guest molecules.

**Figure 3:**
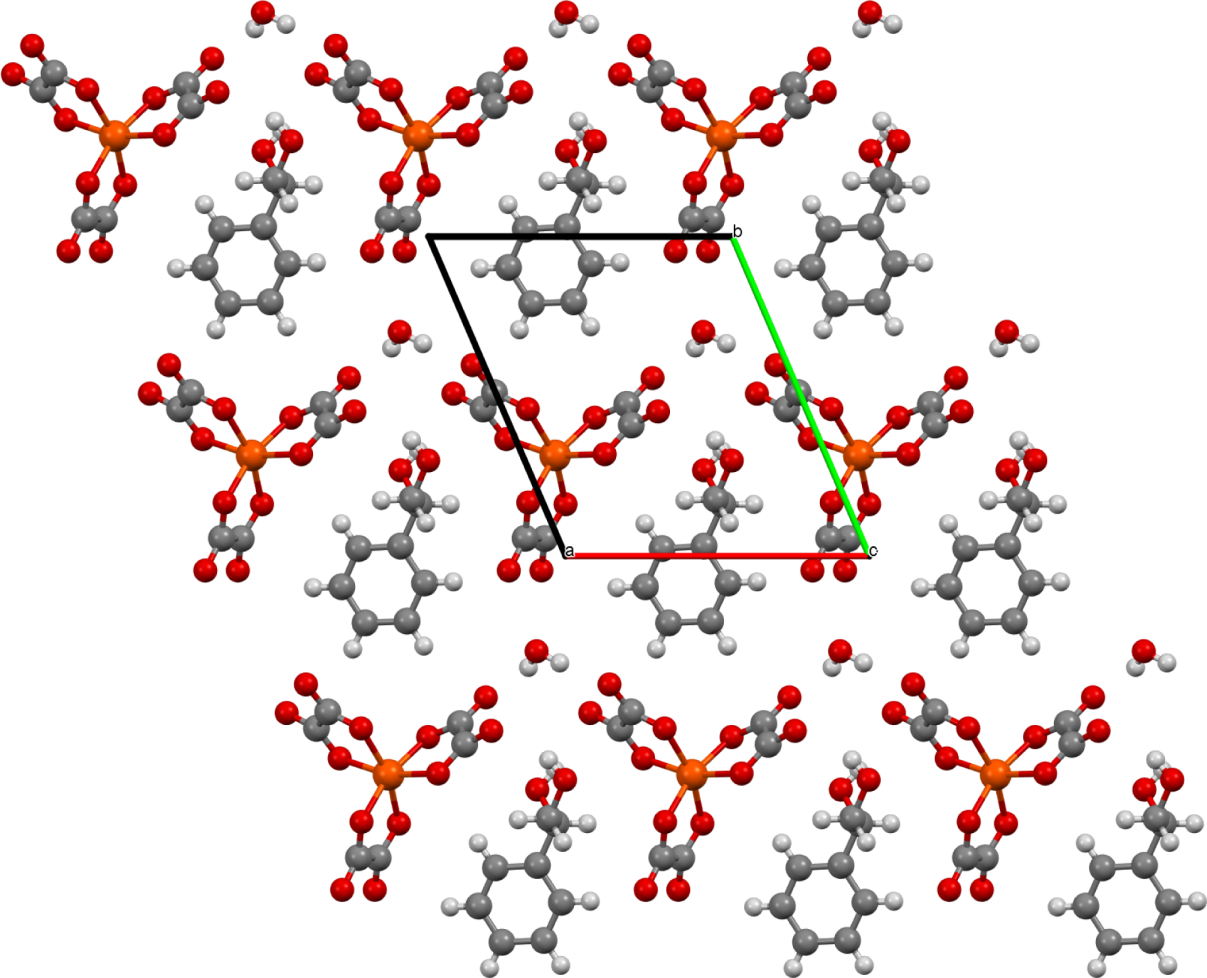
Anion layer of salt (**I**) viewed down the *c* axis.

The donors and anions form segregated layers (Figure 1) with two unique donor layers, each sandwiched between centrosymmetrically related anion layers. The BEDT-TTF donors have a β” packing arrangement with the two crystallographically independent donors within each β’’ layer adopting an···AABBAA··· packing order within a stack, with the donors within the pairs related by a center of symmetry (Figure 2). The two donor layers show different beta’’ packing arrangements (Figure 2). Donor layer 1 has only side-to-side S···S contacts, while Donor layer 2 also has diagonal S···S interactions between donors (Table 1). Donor layer 1 (Figure 2 left) is on the edge of the unit cell (*c* axis), docking into anion layers and facing the primary −CH_2_OH groups of the guest molecules. Donor layer 2 (Figure 2 right) is in the center of the unit cell (*c* axis) and docks into anion layers facing the phenyl groups of the guest molecule. This type of a bilayered 4:1 salt has been seen before when large nonsymmetrical guest molecules have been used, which protrude more on one side of the anion layer than the other side.^15^ The anion layer (Figure 3) is a hexagonal arrangement of tris(oxalato)ferrate anions and H_2_O/H_3_O^+^ with a 1-phenylethane-1,2-diol guest in the hexagonal cavity. The guest makes two OH···O hydrogen bonds to the outer oxygen atoms of two tris(oxalato)ferrate anions. Figure 1 shows how the guest is sited within the anion layer, presenting a different part of the guest molecule to each of Donor layers 1 and 2. These two different faces of the anion layer lead to two different donor packing arrangements on either side of the anion layer. This has previously produced alpha-beta’’ and kappa-beta’’ salts before, but this is the first example of a β’’-β’’ salt.^15,18^

**Table 1:** Short S···S Contacts (<Sum VdW Radii) in (**I**)

S atom 1···S atom 2	Contact/ Å
S14···S10	3.479(2)
S11···S7	3.457(2)
S9···S7	3.346(2)
S1···S15	3.270(2)
S3···S15	3.370(12)
S2···S6	3.439(2)
S2···S8	3.465(2)
S18···S24	3.514(2)
S24···S29	3.528(2)
S24···S31	3.586(3)
S19···S31	3.474(2)
S23···S25	3.553(2)
S17···S31	3.346(2)
S26···S30	3.5038(19)

The BEDT-TTF donor charges (Table 2) have been estimated as +0.76 and +0.68 in Donor layer 1, and +0.59 and +0.65 in Donor layer 2. All four crystallographically independent donors would be expected to bear a charge of +0.5 if the cation H_3_O^+^ was present—similar to other β’’ salts in this series.^15^ This suggests an overall charge on the four donors of +2.68 augmented by a H_2_O:H_3_O^+^ ratio of 2:1.

**Table 2:**
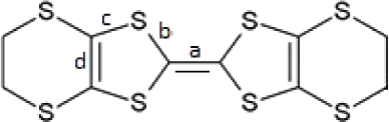
Calculation of the Approximate Charge (*Q*) of BEDT-TTF Molecules in (**I**) from Bond Lengths (Å): δ = (*b* + *c*) – (*a* + *d*), *Q* = 6.347 – 7.463δ^22^

**Donor**	***a***	***b***	***c***	***d***	**δ**	***Q***
A	1.376	1.738	1.747	1.360	0.749	+0.76
B	1.369	1.740	1.751	1.363	0.760	+0.68
C	1.369	1.744	1.754	1.357	0.772	+0.59
D	1.366	1.739	1.748	1.357	0.764	+0.65

**Figure 4:**
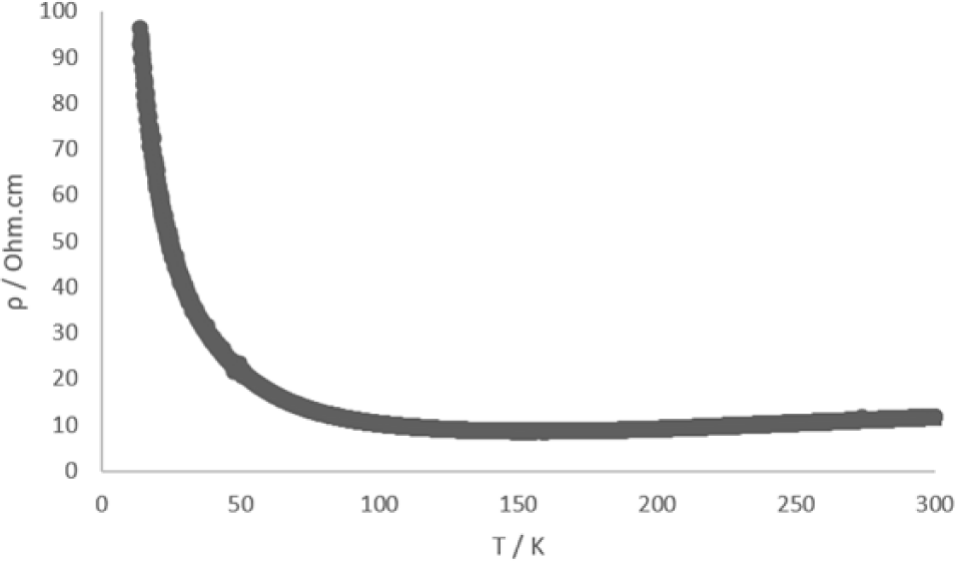
Plot of resistivity versus temperature for salt (**I**).

**Figure 5:**
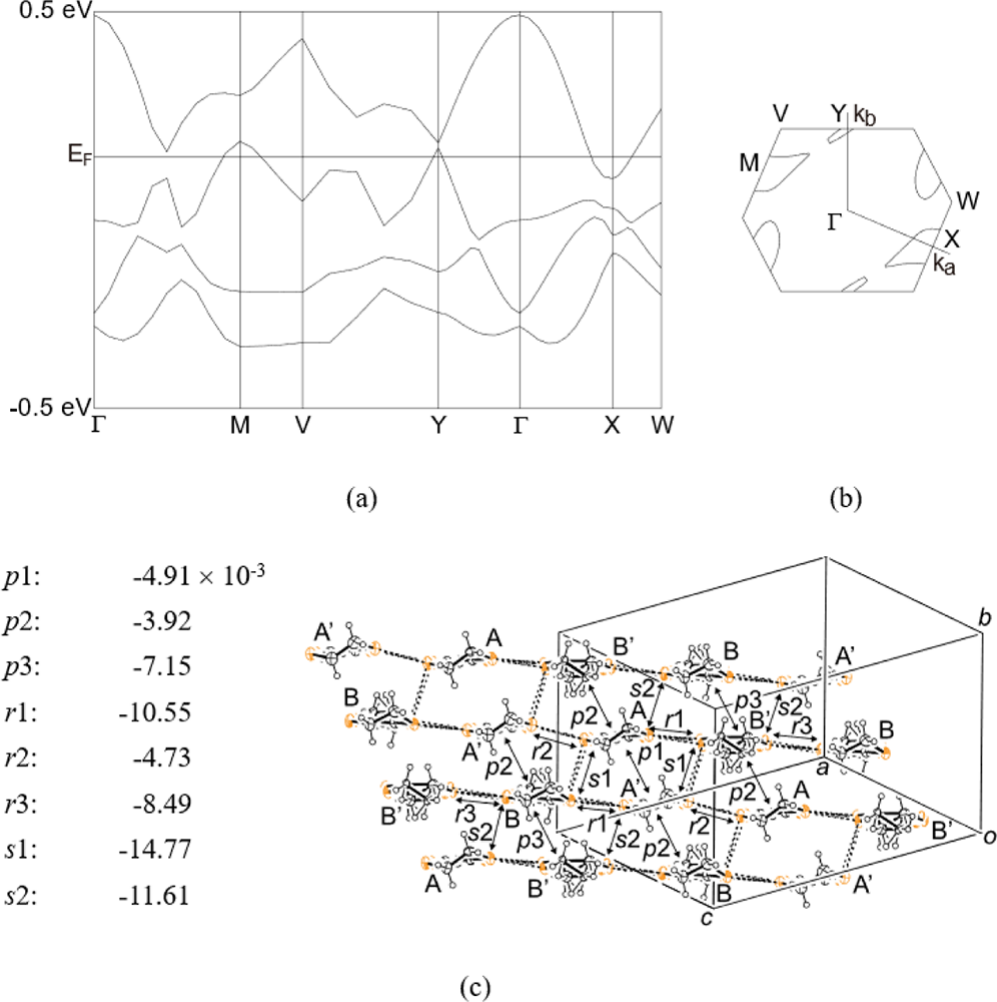
Band dispersions (a), Fermi surfaces (b), and a structure of Donor layer 1 for salt **I** (c). Intermolecular interactions are labeled with the transfer integrals listed in (c).

**Figure 6:**
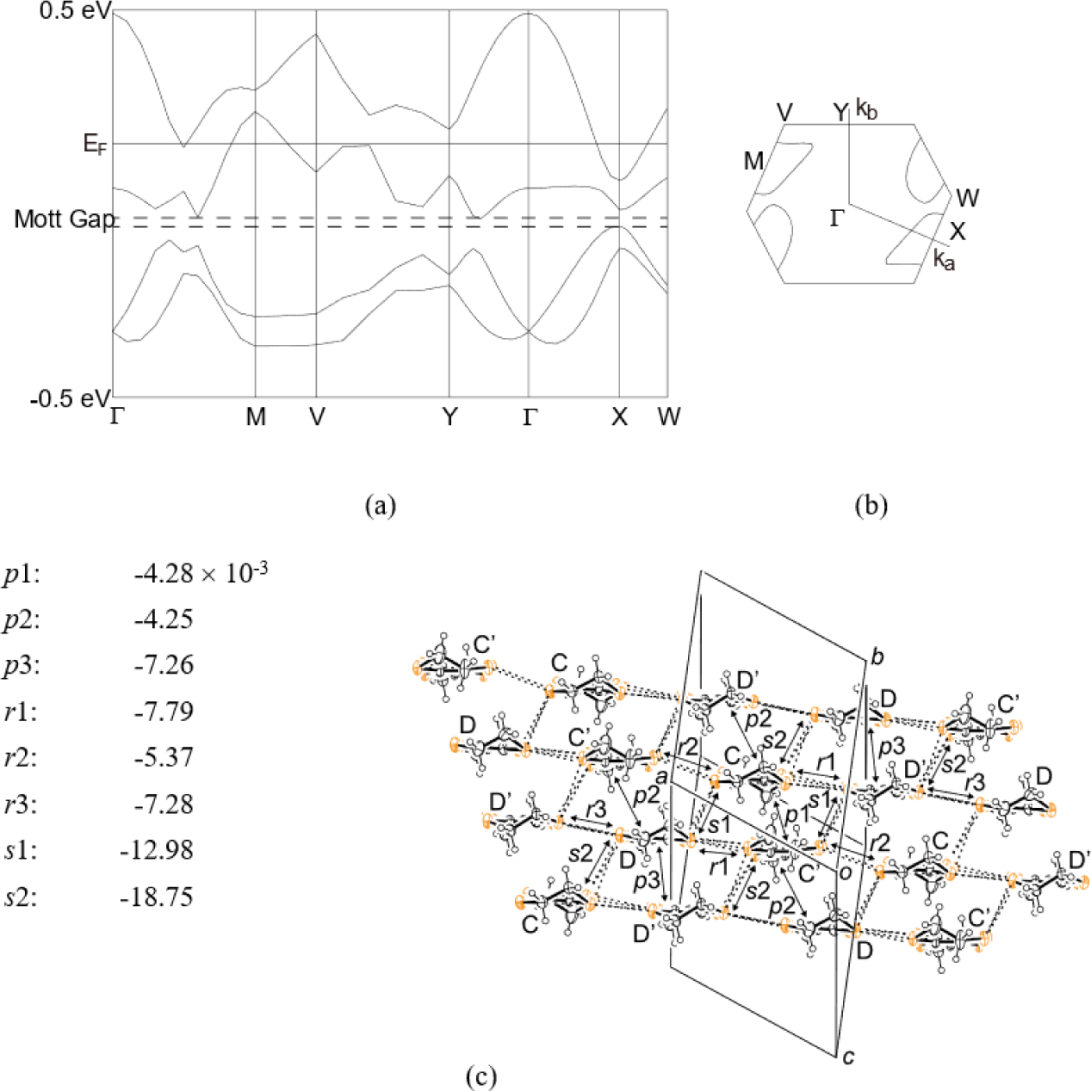
Band dispersions (a), Fermi surfaces (b), and a structure of the Donor layer 2 for salt **I** (c). Intermolecular interactions are labeled with the transfer integrals listed in (c).

Electrical resistivity has been measured on single crystals of this salt, which shows metallic behavior from room temperature (σ_RT_ = 0.098 S cm^–1^) down to 160 K, below which there is a gradual increase in the resistivity (Figure 4). SQUID magnetometry was performed on a polycrystalline sample down to 1.8 K, but no Meissner signal was observed. The Fermi surfaces of Donor layer 1 (Figure 5), which consist of one hole and two electron pockets, are similar to those of the superconducting β’’-(BEDT-TTF)_4_[(H_3_O)Cr(C_2_O_4_)_3_]·PhNO_2_ salt (Cr/PhNO_2_),^20^ with one electron pocket being much smaller than the other. However, the Cr/PhNO_2_ salt has a bandwidth of 0.875 eV, which is approximately 5% larger than 0.831 eV of that in Donor layer 1 of salt (**I**). Applying positive static or chemical pressure to (**I**) increases the bandwidth to provide almost the same Fermi surface as that of the superconducting Cr/PhNO_2_ salt. The Fermi surfaces of Donor layer 2 (Figure 6) resemble those of the superconducting β’’-(BEDT-TTF)_4_[(H_3_O)Ga(C_2_O_4_)_3_]·PhNO_2_ salt (Ga/PhNO_2_).^21^ However, the Ga/PhNO_2_ salt does not have a Mott gap, but Donor layer 2 in salt (**I**) has a Mott gap of 0.020 eV. The upper and lower bandwidths of Donor layer 2 of 0.531 and 0.307 eV are 0.035 (6.2%) and 0.012 eV (3.8%) smaller than those of the Ga/PhNO_2_ salt, which are 0.566 and 0.319 eV, respectively. The narrower bandwidths of Donor layer 2 in salt (**I**) might make the salt nonsuperconducting.

### α-(BEDT-TTF)_5_[Δ-Fe(C_2_O_4_)_3_].[l-(+)-tartaric acid]_2_ (II)

**Scheme 3:**
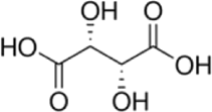
l-(+)-Tartaric Acid

The use of l-(+)-tartaric acid (Scheme 3) as the solid additive in the electrocrystallization medium produces a chiral radical-cation salt of formula α-(BEDT-TTF)_5_[Δ-Fe(C_2_O_4_)_3_].[l-(+)-tartaric acid]_2_ (**II**).

**Figure 7:**
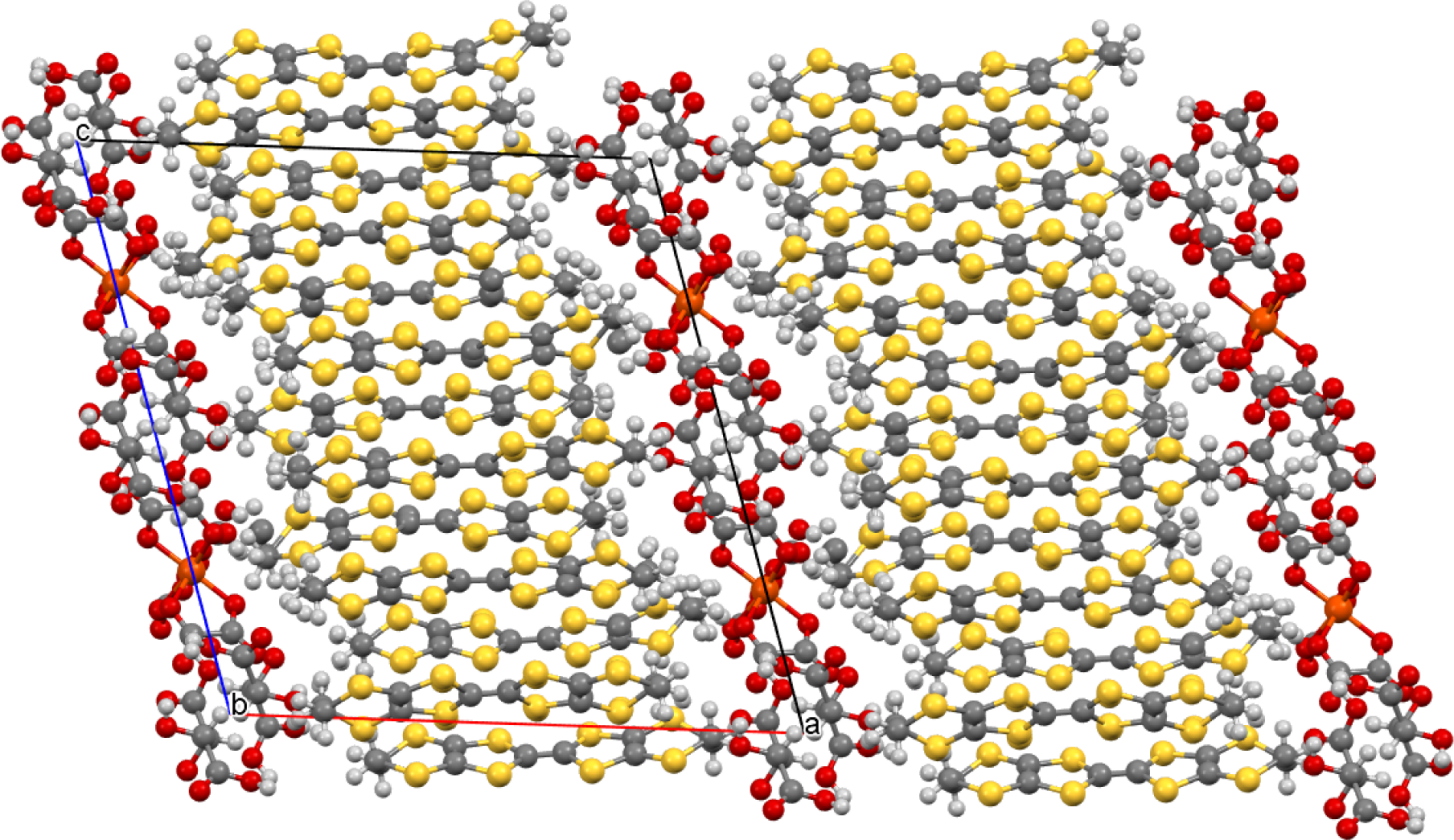
Layered packing of (**II**) viewed down the *b* axis.

α-(BEDT-TTF)_5_[Δ-Fe(C_2_O_4_)_3_].[l-(+)-tartaric acid]_2_ (**II**) crystallizes in the space group *P*2_1_ with a Flack parameter of 0.027(9). This radical-cation salt has an asymmetric unit comprising five crystallographically independent BEDT-TTF donor molecules, an Δ-Fe(oxalate)_3_ anion, and two l-(+)-tartaric acid molecules. The donors and anions form segregated stacks (Figure 7).

**Figure 8:**
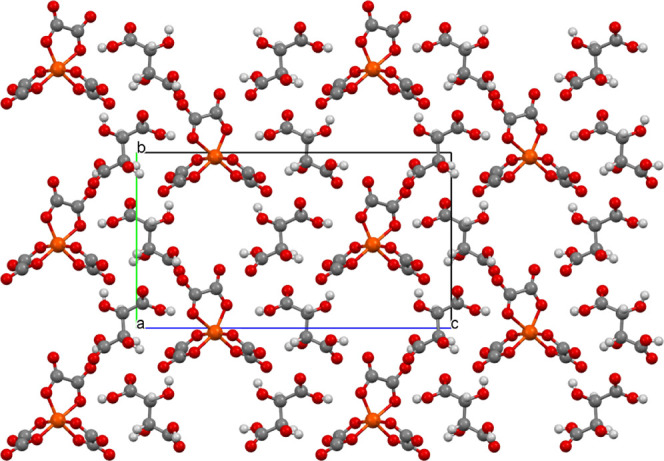
Salt (**II**) anion layer viewed down the *a* axis.

The anion layer (Figure 8) consists of a novel packing of Fe(oxalate)_3_ anions with l-(+)-tartaric acid molecules. Rows of Δ-Fe(oxalate)_3_ and l-(+)-tartaric acid alternate along the *c* axis. The Δ-Fe(oxalate)_3_ anions are located centrally within the anion layer, while l-(+)-tartaric acid molecules are located in either direction along the *a* axis on both faces of the anion layer. All four OH groups of each tartaric acid are involved in hydrogen bonding to tris(oxalato)ferrate anions. Adjacent molecules of l-(+)-tartaric acid along the *c* axis alternate between the opposing faces of the anion layer (Figure 8). Spontaneous resolution of the Fe(oxalate)_3_ anions has occurred with only the Δ enantiomers of Fe(oxalate)_3_ present in the BEDT-TTF radical-cation salt despite starting from a racemic solution of labile Δ/Λ-Fe(oxalate)_3_ (Scheme 1).

**Figure 9:**
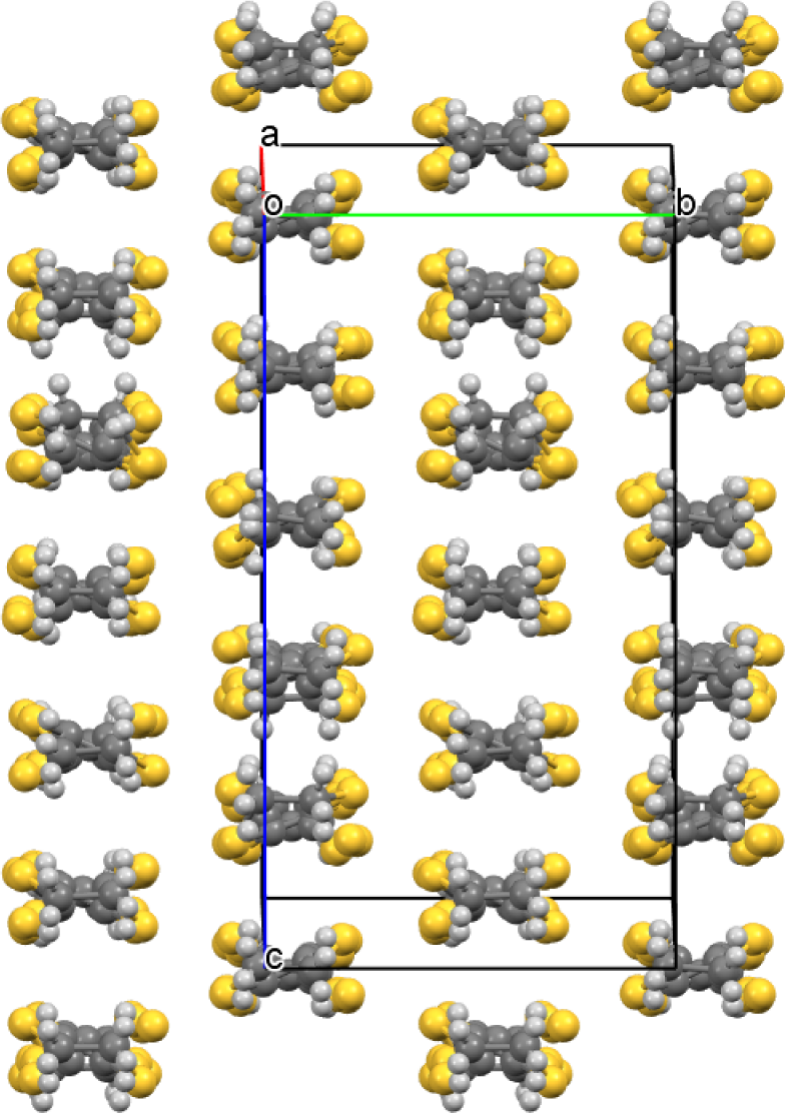
Donor layer of (**II**) showing BEDT-TTF disorder over two positions along the long axis of the molecules.

The BEDT-TTF molecules are disordered about their long axis between two positions (71:29) whose best planes lie at 48–50° (Figure 9). Owing to this disorder, the donor packing motif of any one donor layer could be α or β’’ depending on the orientation of neighboring stacks of BEDT-TTF in the *b* direction. However, if the orientation of neighboring donor stacks is the same (β’’) in the *b* direction, this would lead to unreasonably short S···S contacts. X-ray data on the same crystals were refined in the space group *C*2 having an *a* axis of 77.090(4) Å (vs 20.2195(8) Å in *P*2_1_) with similar *b* and *c* cell lengths to the *P*2_1_ structure, and with a difference in the monoclinic angle of 92.076(7)° (vs 106.900° in *P*2_1_). Refinement showed four crystallographically independent donor layers, with each donor layer having α-type packing, but the data set is of low quality.

**Figure 10:**
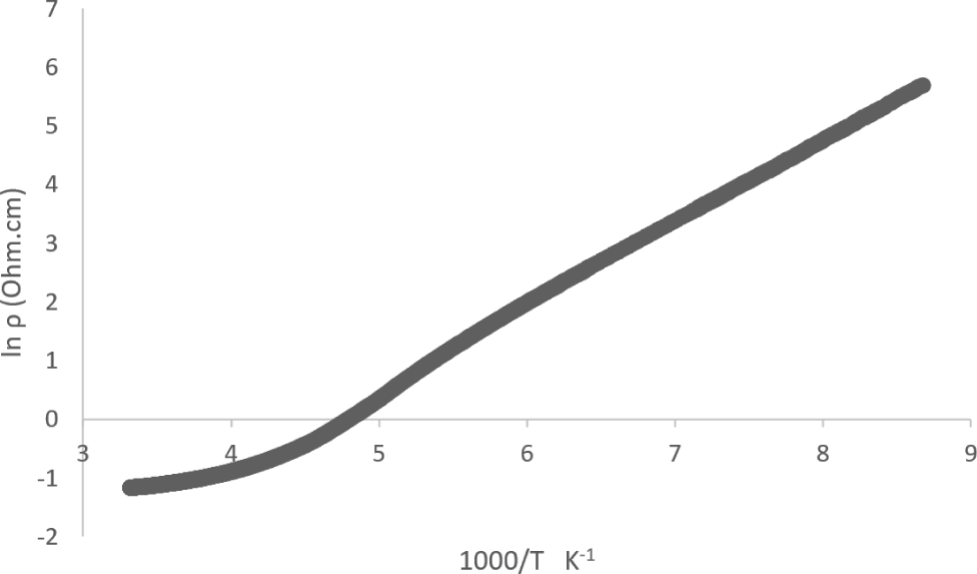
Plot of ln(resistivity) versus 1000/*T* (**II**).

Electrical resistivity has been measured on single crystals of this salt, which shows semiconducting behavior (Figure 10) with a conductivity value at room temperature of 3.210 S cm^–1^ and an activation energy of 0.124 eV below 200 K.

Attempts to prepare the corresponding salts using d-(−)- and racemic tartaric acid for comparison of their electrical properties with those of **II** to observe the influence of the chirality of the additive have not yielded crystals.

### α-(BEDT-TTF)_12_[Fe(C_2_O_4_)_3_]_2_.(H_2_O)_16_.ethanol.(*R*)-(−)-3-hydroxytetrahydrofuran (III)

**Scheme 4:**
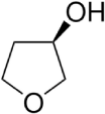
(*R*)-(−)-3-Hydroxytetrahydrofuran

The use of (*R*)-(−)-3-hydroxytetrahydrofuran (Scheme 4) as the additive in the electrocrystallization medium produces a chiral radical-cation salt of formula α-(BEDT-TTF)_12_[Fe(C_2_O_4_)_3_]_2_.(H_2_O)_16_.ethanol.(*R*)-(−)-3-hydroxytetrahydrofuran (**III**). This is a remarkable crystal structure containing 32 independent molecular species in the asymmetric unit.

Salt (**III**) crystallizes in the space group *P*2_1_ with a Flack parameter of 0.034(6). This radical-cation salt has an asymmetric unit of 12 crystallographically independent BEDT-TTF donor molecules, two Fe(oxalate)_3_ anions, 16 water molecules, one ethanol molecule, and one (*R*)-(−)-3-hydroxytetrahydrofuran molecule. A 12:2 radical-cation salt has been observed previously in the salt (BEDT-TTF)_12_[Fe(C_2_O_4_)_3_]_2_.15 or 16H_2_O.^23^

**Figure 11:**
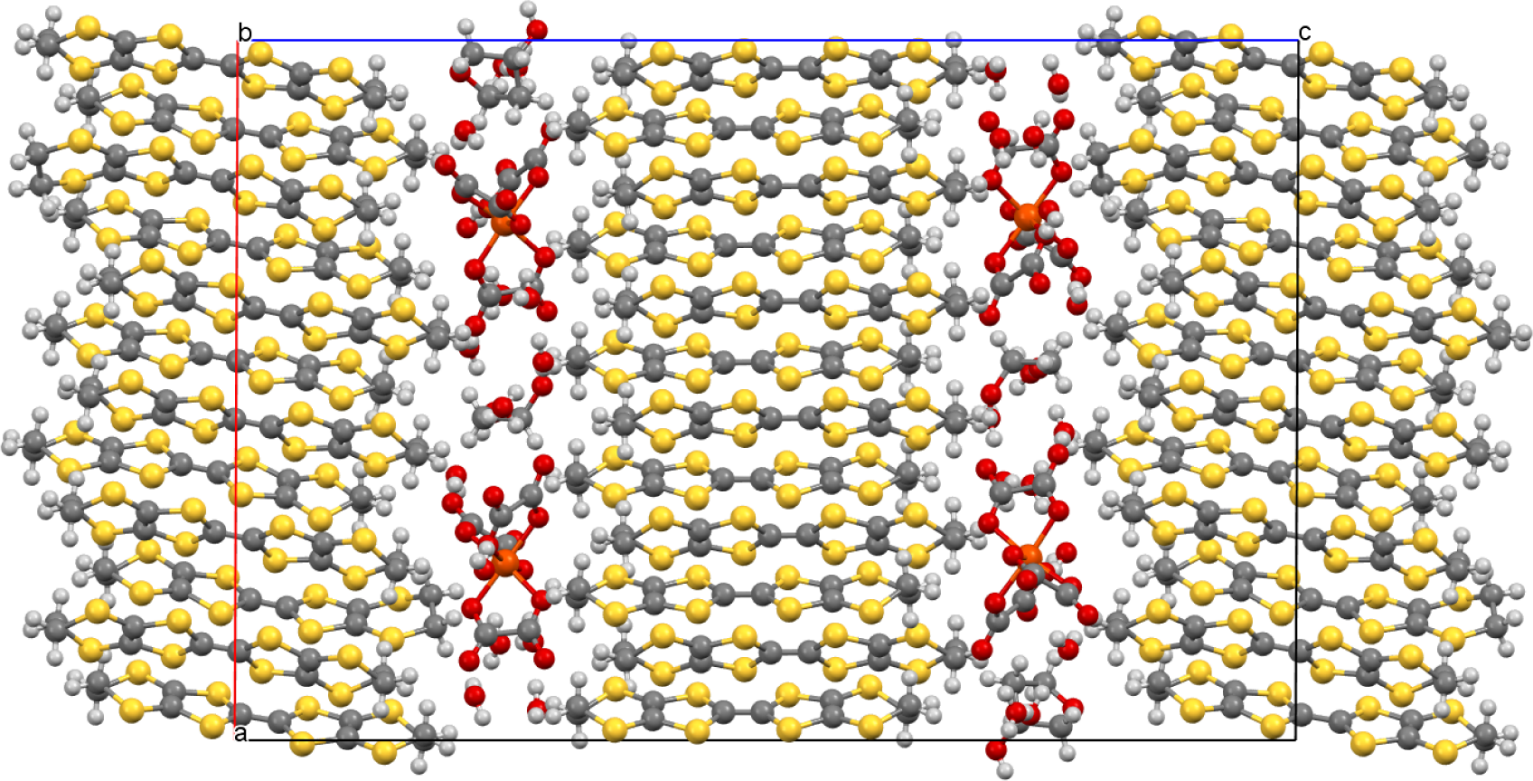
Layered structure of (**III**) viewed down the *b* axis.

**Figure 12:**
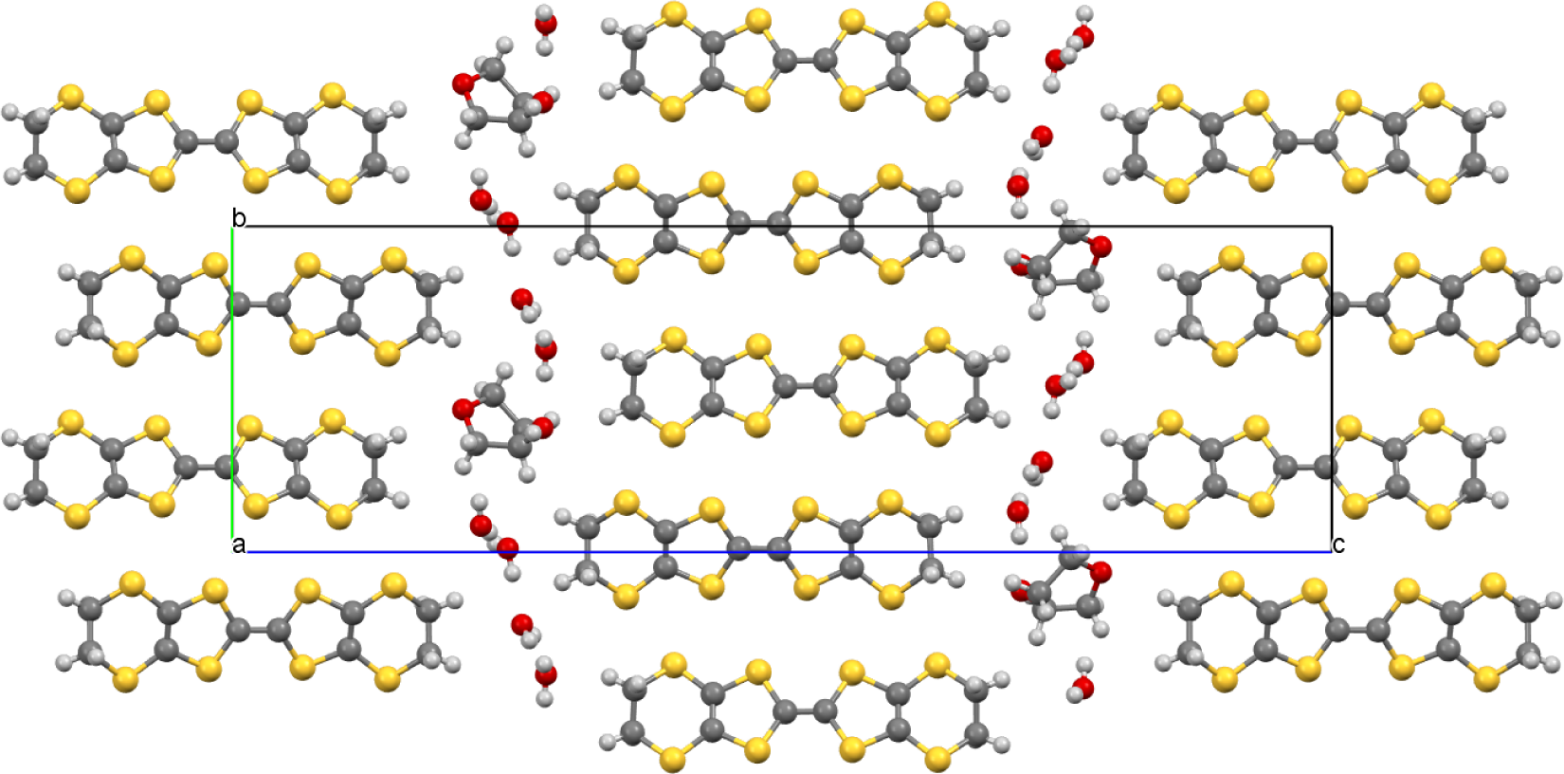
Layered structure of (**III**) viewed down the *a* axis, showing only the water and guest THF molecules in the anion layer.

**Figure 13:**
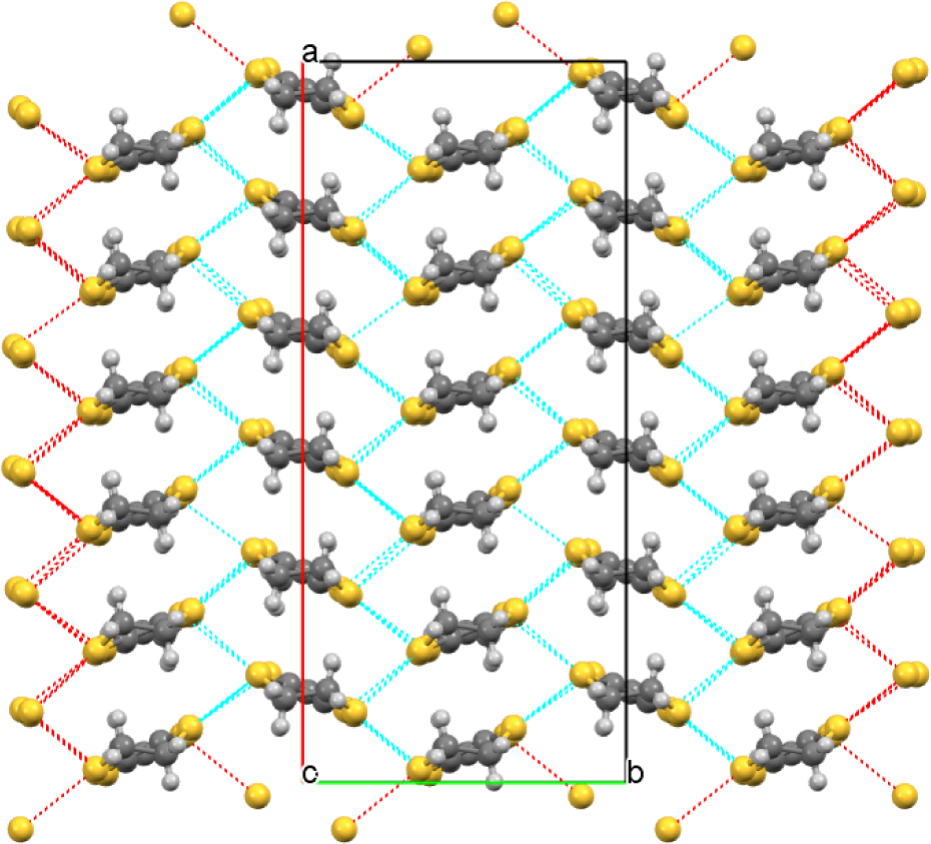
Donor layer at the center of the unit cell in (**III**) showing short S···S contacts.

**Figure 14:**
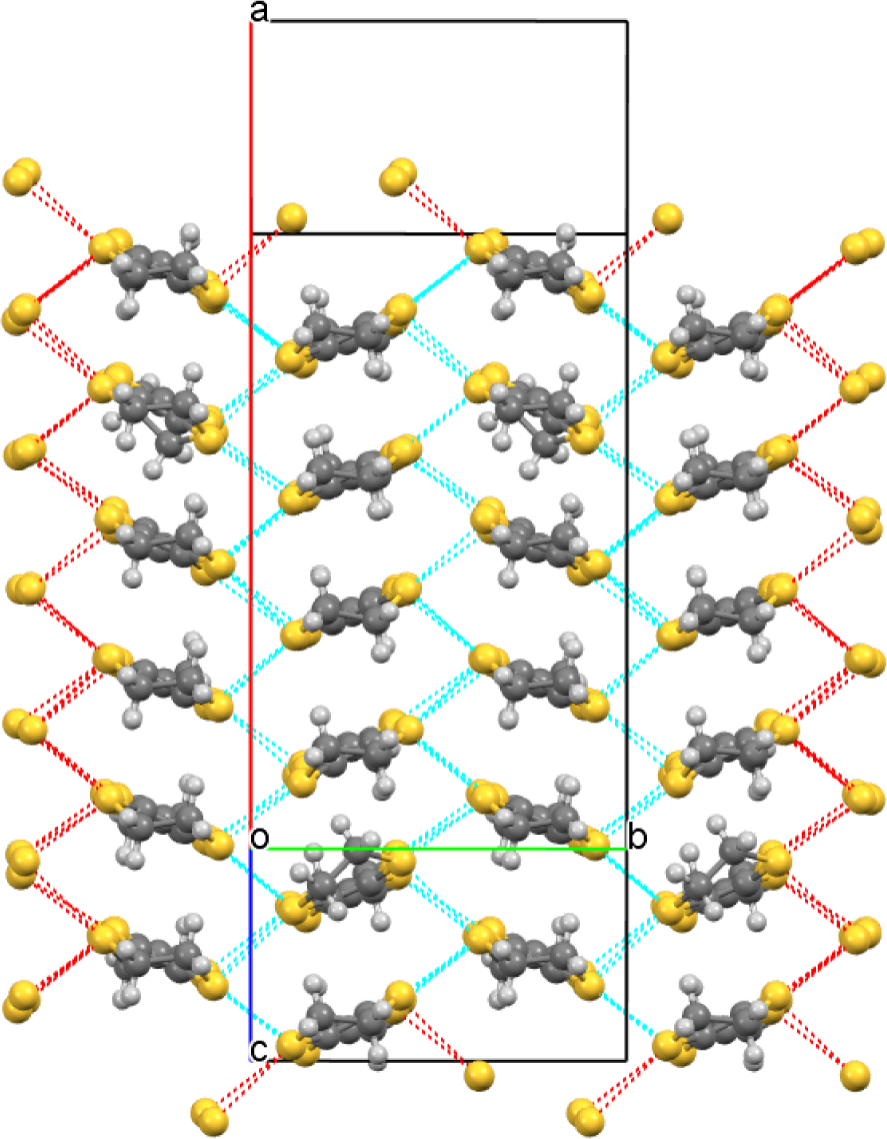
Donor layer at the end of the unit cell in (**III**) showing short S···S contacts.

**Figure 15:**
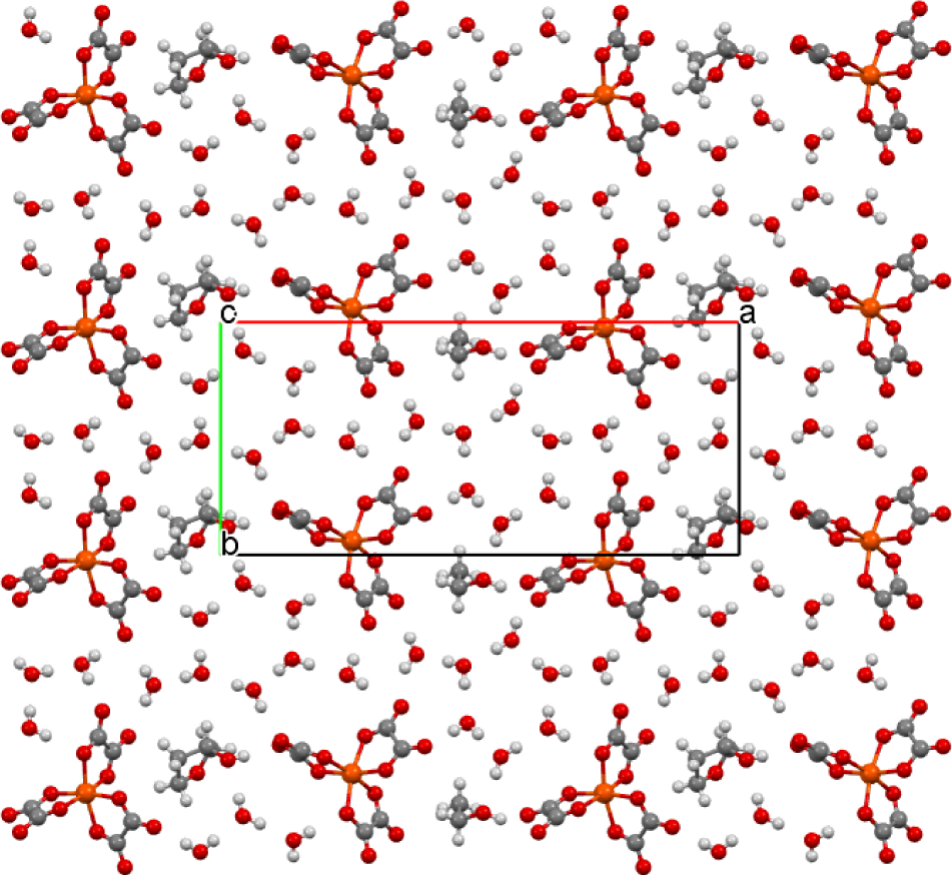
Anion layer of (**III**) viewed down the *c* axis.

**Figure 16:**
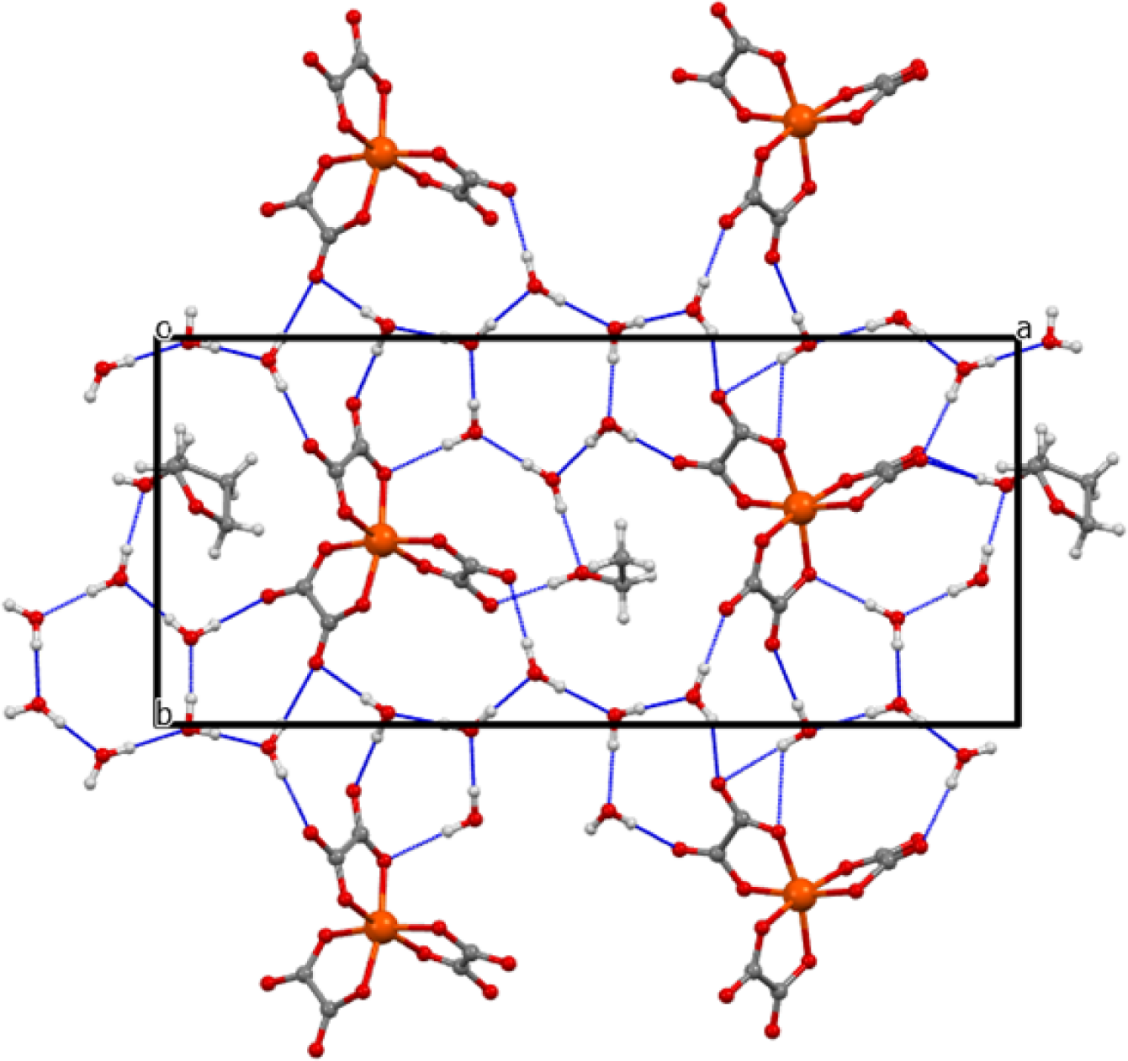
Hydrogen bonding network in the anion layer of (**III**) viewed down the *c* axis.

The donors and anions form segregated stacks (Figures 11 and 12) with the BEDT-TTF donors having alpha packing (Figures 13 and 14) with a number of side-to-side S···S close contacts. There are two crystallographically independent donor layers, one at the center and the other at the end of the *c* axis of the unit cell, each containing six unique donor molecules. The anion layer (Figure 15) consists of a novel packing of Fe(oxalate)_3_ anions with 16 waters, an ethanol, and (*R*)-(−)-3-hydroxytetrahydrofuran molecules. Fe(oxalate)_3_ is present in both enantiomeric forms, with rows of Δ and Λ isomers alternating along the *a* axis. The hydrogen bonding network within the anion layer is shown in Figure 16 and involves the hydroxyl groups of the chiral additive and ethanol. Of the 16 water molecules, 12 are included in two hexagons connected by hydrogen bonding.

**Figure 17:**
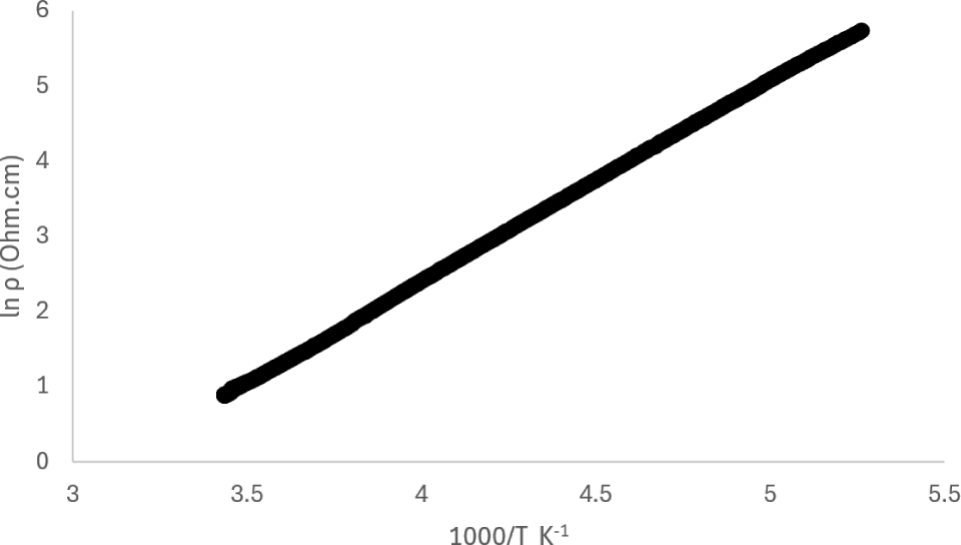
Plot of ln(resistivity) versus 1000/*T* for (**III**).

S···S close contacts and BEDT-TTF charge estimations based on bond lengths will not be discussed in detail owing to the high *R* factor of the X-ray data and the large number of independent BEDT-TTF molecules. Electrical resistivity has been measured on single crystals of this salt, which shows semiconducting behavior (Figure 17) with a conductivity value at room temperature of 0.412 S cm^–1^ and an activation energy of 0.229 eV.

## Conclusions

We have demonstrated that the inclusion of an enantiopure guest in the preparation of radical-cation salts from BEDT-TTF and tris(oxalato)ferrate by electrocrystallization can lead to a variety of outcomes, which may be difficult to predict beforehand. Of most importance, in the presence of enantiopure l-(+)-tartaric acid, we observe spontaneous resolution of the labile tris(oxalato)ferrate to produce a chiral radical-cation salt of BEDT-TTF, whose asymmetric unit includes the chiral guest molecule, five α-(BEDT-TTF) donors, [Δ-Fe(C_2_O_4_)_3_], and two l-(+)-tartaric acid molecules. Using enantiopure (*R*)-(−)-3-hydroxytetrahydrofuran gives a complex salt with 32 unique species α-(BEDT-TTF)_12_[Fe(C_2_O_4_)_3_]_2_.(H_2_O)_16_.(ethanol).(*R*)-(−)-3-hydroxytetrahydrofuran, which includes the chiral guest and contains a racemic mixture of Λ and Δ enantiomers of Fe(C_2_O_4_)_3_. Using enantiopure (*S*)-phenyloxirane, the chiral additive has hydrolyzed to racemic 1-phenylethane-1,2-diol to produce a racemic metallic salt β”-β”-(BEDT-TTF)_4_(H_2_O/H_3_O)Fe(C_2_O_4_)_3_.1-phenylethane-1,2-diol, which has two crystallographically independent β” layers. We are currently preparing the chiral forms of this salt from (*S*)-(+)- and (*R*)-(−)-1-phenylethane-1,2-diol to investigate eMChA.

## Experimental Details

### Starting Materials

Ammonium tris(oxalato)ferrate trihydrate, 1,2,4-trichlorobenzene, l-(+)-tartaric acid, (*S*)-phenyloxirane, ethanol, and 18-crown-6 were purchased from Sigma-Aldrich and used as received. BEDT-TTF was purchased from TCI and recrystallized from chloroform. (*R*)-(−)-3-Hydroxytetrahydrofuran was purchased from Fluorochem and used as received.

### Synthesis of Radical-Cation Salts

Salts (**I**) and (**II**) were synthesized by adding 100 mg of ammonium tris(oxalato)ferrate, 250 mg of 18-crown-6, and 100 mg of (*S*)-phenyloxirane (**I**), or 100 mg of l-(+)-tartaric acid (**II**) to a mixture of 15 mL of 1,2,4-trichlorobenzene and 3 mL of ethanol and stirring overnight before filtering into a H cell containing 10 mg of BEDT-TTF in the anode side, and a current of 0.5 μA was applied for 8 weeks to give black blocks.

Salt (**III**) was synthesized by adding 150 mg of ammonium tris(oxalato)ferrate, 450 mg of 18-crown-6, and 1.8 mL of (*R*)-(−)-3-hydroxytetrahydrofuran to a mixture of 15 mL of 1,2,4-trichlorobenzene and 3 mL of ethanol and stirring overnight before filtering into a H cell containing 10 mg of BEDT-TTF on the anode side. Five drops of water were added to the anode side of the H-cell, and a current of 1.0 μA was applied for 7 weeks to give a cluster of small black plates.

18-crown-6 is included to aid the dissolution of the ammonium salt of the tris(oxalato)ferrate. Platinum electrodes of 1 cm length and 0.40 mm diameter were used for the electrocrystallization.

## Electrical Resistivity Measurements

Temperature-dependent electrical resistivity measurements were performed using four contacts on single crystals of **I–III**.

### X-RAY CRYSTALLOGRAPHY

Data for (**I**) and (**III**) were collected on a Rigaku XtaLAB Synergy DW system with a HyPix-Arc 100 detector using a Cu source at 120 K. Data for (**II**) were collected on a Rigaku Oxford Diffraction Xcalibur system equipped with a Sapphire detector using a Cu source at 150 K. For salt **II**, the occupancy of the major orientation of the donors was refined to 0.710(2).

For salt (**III**) five atoms (C1A, C5A, C4B, C6C, and C8F) had to be left isotropic. Hydrogens belonging to the 16 water molecules were placed to make a reasonable hydrogen bonding network between each other and other −OH groups and acceptor O atoms. These positions were not refined. O···O distances are in the range of 2.67–2.89 Å.

All data were collected using CrysAlisPro^24^ and then solved and refined using SHELXT^25^ within OLEX2.^26^ Images were produced using Mercury.^27^

#### Crystal Data for (**I**)

C_54_H_44_FeO_15_S_32_, *M* = 2014.66, black prism, *a* = 9.6581(2), *b* = 10.9638(3), *c* = 38.0447(8) Å, α = 89.295(2)°, β = 82.890(2)°, γ = 66.880(2)°, *U* = 3673.58(16) Å^3^, *T* = 120 K, space group *P*1̅, *Z* = 2, μ = 10.697 mm^–1^, reflections collected = 82840, independent reflections = 15110, *R*1 = 0.0883, *wR*2 = 0.2352 [*F*^2^ > 2σ(*F*^2^)], *R*1 = 0.1078, and *wR*2 = 0.2544 (all data).

#### Crystal Data for (**II**)

C_64_H_52_Fe_1_O_24_S_40_, *M* = 2543.30, black block, *a* = 20.2195(8), *b* = 11.1931(4), *c* = 20.9585(9) Å, β = 106.900(5)°, *U* = 4538.5(3) Å^3^, *T* = 120 K, Flack parameter = 0.027(9), space group *P2*_*1*_, *Z* = 2, μ = 10.581 mm^–1^, reflections collected = 64145, independent reflections = 16466, *R*1 = 0.0866, *wR*2 = 0.2345 [*F*^2^ > 2σ(*F*^2^)], *R*1 = 0.1448, *wR*2 = 0.2812 (all data).

#### Crystal Data for (**III**)

C_138_H_142_Fe_2_O_43_S_96_, *M* = 5677.97, black block, *a* = 24.8553(4), *b* = 11.1595(2), *c* = 37.7064(6) Å, β = 90.250(1)°, *U* = 10458.6(3) Å^3^, *T* = 120 K, space group *P2*_*1*_, *Z* = 2, μ = 10.571 mm^–1^, reflections collected = 166351, independent reflections = 38969, *R*1 = 0.1173, *wR*2 = 0.3163 [*F*^2^ > 2σ(*F*^2^)], *R*1 = 0.1379, *wR*2 = 0.3362 (all data).
